# Oxytocin Increases Generosity in Humans

**DOI:** 10.1371/journal.pone.0001128

**Published:** 2007-11-07

**Authors:** Paul J. Zak, Angela A. Stanton, Sheila Ahmadi

**Affiliations:** 1 Center for Neuroeconomics Studies and Department of Economics, Claremont Graduate University, Claremont, California, United States of America; 2 Department of Neurology, Loma Linda University Medical Center, Loma Linda, California, United States of America; 3 Argyros School of Business & Economics, Chapman University, Orange, California, United States of America; 4 Division of Endocrinology, Geffen School of Medicine, University of California Los Angeles, Los Angeles, California, United States of America; Georgia State University, United States of America

## Abstract

Human beings routinely help strangers at costs to themselves. Sometimes the help offered is generous—offering more than the other expects. The proximate mechanisms supporting generosity are not well-understood, but several lines of research suggest a role for empathy. In this study, participants were infused with 40 IU oxytocin (OT) or placebo and engaged in a blinded, one-shot decision on how to split a sum of money with a stranger that could be rejected. Those on OT were 80% more generous than those given a placebo. OT had no effect on a unilateral monetary transfer task dissociating generosity from altruism. OT and altruism together predicted almost half the interpersonal variation in generosity. Notably, OT had twofold larger impact on generosity compared to altruism. This indicates that generosity is associated with both altruism as well as an emotional identification with another person.

## Introduction

Human beings show considerable generosity toward strangers. In 2005, over $260 billion was given to U.S. charities, with $199 billion (77%) of this given by individuals [1]. The absolute amount of charitable giving is not only high, but the proportion of income donated has grown. In 1954, the average individual in the U.S. gave 1.9% of after-tax income to charity ($222), while in 2005 giving averaged 2.2% of after-tax income ($656, inflation adjusted) [1]. In 2005, approximately one-third of this giving was directed to religious organizations, followed by 19% to health and human services, and 15% to education. People give of their time as well as money. In 2005, over 65 million Americans volunteered to help charities [1]. Ninety-six percent of volunteers said that one of their motivations was “feeling compassion toward other people” [2, p 7]. In the midst of all this giving, the physiologic mechanisms that support altruism and generosity are little understood.

Several evolutionary mechanisms have been proposed to explain altruistic giving. These include kin selection, direct and indirect reciprocity, group or multi-level selection, and strong reciprocity. Kin selection [3]–[4] does not explain all altruistic giving because a proportion of giving is to nonrelatives. A recent study found that an average of 1.3% of household income was given to nonrelatives (roughly the same amount given to religious organizations), and an average of 20.3 person-days was spent helping nonrelatives during a year [5]. Reciprocal altruism is giving with an expectation of equal or larger future return from the same person. Yet much charitable giving and direct helping of others does not appear to provide direct reciprocation, for example, volunteering or donating blood [2]. Indirect reciprocity is giving to one person in the expectation of return from another [6]–[8]. This relies on reputation and does not explain anonymous giving [9] that is the focus of this paper. Group selection can support altruistic giving to nonkin as an evolutionarily stable strategy if individuals can be excluded from the group [10]–[12]. Exclusion is difficult when giving to large organizations like the Red Cross, even though much giving is in-group directed [2]. Another multi-level selection theory, strong reciprocity, was recently proposed to explain altruistic acts [13]–[14]. Strong reciprocity, defined as altruistically rewarding cooperators and punishing defectors, does not explain generosity when resources are scarce. Indeed, none of these evolutionary models explicitly predicts generosity in anonymous one-shot interactions.

In this paper we investigate a mechanism that may produce generosity while dissociating generosity from altruism. Altruism is defined as helping another at a cost to oneself [Sober, p 17, 15]. Generosity is defined as “liberality in giving” [16] or offering more to another than he or she expects or needs. Generosity is therefore a subset of altruism. For example, one may give a homeless person 25 cents (altruism) or ten dollars (altruism and generosity).

The role of empathy in prompting altruistic acts has been proposed by behavioral scientists [17]–[23], though the roots of this idea come from Thomas Aquinas (1225-1274)[24], David Hume (1711-1776) [25], and Adam Smith (1723-1790) [26]. Neuroimaging experiments in humans measuring empathic responses have revealed activity in a network of brain regions, including areas that process emotional and social information, premotor regions, as well as pain pathways. Nonhuman primates have also been shown to exhibit empathy [27], indicating that human empathy has evolutionary roots. Studies measuring human brain activity during charitable giving have shown that giving appears to activate reward regions of the brain, as well as areas associated with emotions and social behaviors [28]–[30]. Coincident brain regions associated with empathy and charitable giving are primarily found in subcoritical areas that process emotional stimuli.

We investigated the role of empathy in producing generosity by manipulating a physiologic mechanism hypothesized to instantiate empathy, the neuromodulator oxytocin (OT). A substantial animal literature has established that OT facilitates attachment to offspring, and in monogamous mammals, cohabiting sexual partners and same-sex conspecifics [31]–[33]. Recent human studies have shown that OT facilitates a temporary attachment between strangers, increasing trust and reciprocity [34]–[38]. In the present paper, we test whether OT is a proximate mechanism prompting generosity between anonymous human strangers. Two tasks were used to dissociate the physiologic role of empathy in producing generosity and altruism using monetary transfers. Monetary transfers were used to obtain objective and active measures of generosity and altruism.

A simple mathematical model will clarify our experimental approach (related models have been proposed [39]–[42], and others). Consider a dyadic interaction between two individuals, *i* and *j*. Let *b_i_* be the benefit that *i* receives, and *b_j_* the benefit to *j*. Individuals obtain utility from receiving their own benefit, and possibly from the other person receiving a benefit. We include a parameter α∈[0,1] that captures the empathy one has for the other person. We use the term empathy in its standard meaning of “an identification with and understanding of another's situation or feelings” [43]. We expect α to be higher when one is induced to explicitly consider one's dyadic partner's feelings regarding the benefits being offered. This has been called “perspective taking” by social psychologists [44].

For simplicity, let utility be given by a standard form *b* ^β^, where β∈(0,1). Let total benefits be limited, b_i_+b_j_ = M<∞. If person *i* is asked to split the *M* benefits between him/herself and person *j*, then *i* faces the following utility maximization problem,
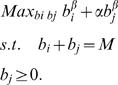
When α = *0*, individual *i* is completely selfish, when α = *1* s/he is egalitarian. It is straightforward to show that *i*'s choice of the benefit offered to *j* increases when α is higher.

Our experimental strategy was to induce participants to consider another's reaction to a split of benefits by giving *j* a chance to punish *i* for a stingy offer. In a separate task, *i* was prompted to make a unilateral monetary transfer to *j* absent punishment. Because a unilateral transfer does not require considering another's perspective, we expected this task to produce a lower α and an associated smaller monetary transfer. In order to demonstrate the causal effect of OT on generosity, we infused one-half of the participants with OT intranasally while the other half received the same amount of normal saline.

Two decision tasks from experimental economics, the ultimatum game (UG) and the dictator game (DG) were used. In both tasks, participants in randomly-formed dyads were assigned the role of decision-maker 1 (DM1), or decision-maker 2 (DM2). In the UG, DM1 was endowed with $10 and was asked to offer a split of this money to DM2. DM2 has no endowment. If DM2 accepted the split, the money was paid. But, if DM2 rejected the split, both DMs earned nothing. Participants were asked to make decisions as both DM1 and DM2, with subsequent random assignment of roles. As DM2s, they were asked to state the minimum amount they would accept from a DM1. The rejection threshold was not reported to the other DMs. By asking subjects for the minimum acceptable offer, the UG task was designed to have participants consider how the DM2 in the dyad would react to an offer (perspective taking). A rejection of DM1's offer in the UG allowed DM2 to punish DM1 for stingy offers, but at a cost.

We define a *generous* transfer in the UG as a DM1 offer that exceeds the average minimum acceptable offer. That is, generous offers are those which are greater than are expected for acceptance.

The DG is similar to the UG in that DM1 has a $10 endowment and DM2 has nothing. The difference in the DG is that DM2 has no choice—he or she must accept whatever DM1 offers. As a result, the DG does not compel DM1 to consider how DM2 will feel about the split of benefits (reduced perspective taking). The consensus view in experimental economics is that the transfer in the DG is a measure of altruism [39], [45]. The inclusion of both the UG and DG allows us to dissociate generosity from altruism within subjects.

In both the UG and DG, subjects whose identities were masked to each other and the experimenters, were randomly assigned to dyads without pre- or post-decision communication. All participants received the same instructions, and there was no deception. Participants were infused with 40 IU oxytocin (OT) or placebo (normal saline) intranasally (see Materials and Methods). The infusion was double-blind. Participants were privately paid their earnings in cash at the conclusion of the experiment. Our approach followed a related study using OT to examine interpersonal trust by means of monetary transfers [37].

## Results

**Figure 1:**
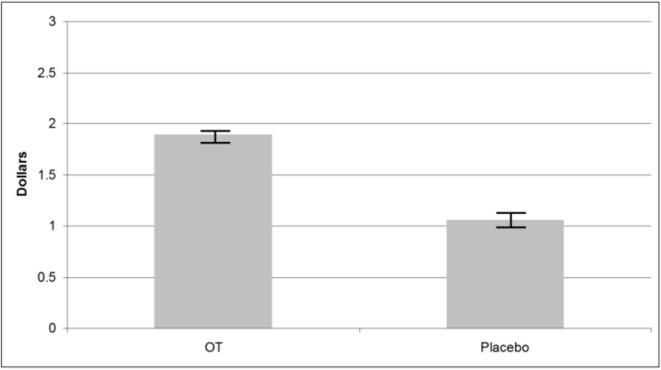
Oxytocin and generosity. Mean DM1 generosity (DM1 UG offer minus average minimum acceptable offer) for those receiving OT or placebo. Generosity is 80% larger in the OT group (p = 0.005, N = 68). The increased generosity is not due to altruism as OT infusion in a task designed to isolated altruism showed no impact relative to placebo (p = 0.51, N = 68).

The mean DM1 offer in the UG was 21% larger in the OT group than in the placebo group (OT mean $4.86 (SD $1.06); placebo mean $4.03 (SD $1.29); two-tailed Mann-Whitney U test p = 0.005; N = 68). Only two subjects (both in the placebo group) offered the subgame perfect Nash equilibrium of $1 in the UG. The DM2 minimum acceptable offer was unaffected by OT (OT mean: $3.03 (SD $1.69); placebo mean: $2.91 (SD $1.74); two-tailed Mann-Whitney, p = 0.78). Aggregating the OT and placebo treatments, the average minimum acceptable offer was $2.97. Using the average minimum acceptable offer, we found that generosity was 80% higher in OT group than in the placebo group (OT mean: $1.89 (SD $1.06); placebo mean: $1.06 (SD $1.29); two-tailed Mann-Whitney U-test p = 0.005; see Fig. 1).

Having a minimum acceptable threshold in the UG means that some offers are rejected. It is possible that subjects on OT were better able to forecast the rejection threshold and thus earned as much or more money than placebo participants, rendering generosity costless. The likelihood of an offer being rejected is nearly identical for both groups (OT percent rejected: 14.3%; placebo percent rejected: 14.7%). The average money earned by the DM1s in the OT group was 5.2% lower than DM1s in the placebo group (OT mean: $4.91 (SD $1.27); placebo mean: $5.18 (SD $1.91); one-tailed Mann-Whitney U-test p = 0.03), showing that generosity in the UG comes with a cost.

The theory presented above predicts that giving to others will be reduced when another's reactions are not explicitly considered. A transfer in the DG provides a measurement of altruism absent taking another's perspective. We found that OT did not impact DM1 transfers in the DG (OT mean: $3.77 (SD $2.21); placebo mean: $3.58 (SD $2.15); two-tailed Mann-Whitney test p = 0.51). As predicted, transfers in the DG were less than in the UG. This held even when comparing the DG transfer to UG transfer in participants who were given the placebo (two-tailed Mann-Whitney test p = 0.04, N = 34).

We also examined whether the effect of OT on generosity in the UG continued to hold after accounting for a participant's altruism in the DG. A least squares regression of generosity in the UG, controlling for the amount of altruism participants revealed in the dictator game, and a binary OT indicator showed that OT continues to be significantly associated with generosity (OT coeff. 0.648, two-tailed t-test p = .014; DG coeff. = .376, two-tailed t-test p = .0001; N = 68, R^2^ = .43).

## Discussion

We have shown that OT raised generosity in the UG by 80% over placebo, and that generous participants left the experiment with less money. The increased generosity was not due to greater altruism because OT did not affect transfers in the DG, and the impact of OT on generosity remains significant even when altruism in the DG was taken into account. This finding is consistent with a recent fMRI study of charitable giving that found evidence for both altruism and “warm glow” motivations for charity [30]. In the present study, OT and altruism together predicted almost half the interpersonal variation in generosity. Notably, our analysis showed that OT has approximately twice the effect on generosity as altruism.

The difference in the decision process between the UG and the DG, shown in the mathematical model above, suggests a reason for these findings. DM1's decision in the UG required a forecast of how the DM2 would react to a proposal because of the threat of punishment. Recent research has shown that stingy offers in the UG provoke negative emotions in DM2s [46], and activate a region of the brain associated with visceral disgust [47]. Our experimental design increased OT in one-half of the participants with the expectation that DM1s would have a more acute understanding of how DM2s would react to an offer in the UG. Because OT receptors in the human brain are preferentially located in areas associated with emotions and social behaviors (especially the amygdala, hypothalamus, and anterior cingulate [48]–[49]), this suggests a role for emotions in supporting generosity. Emotional engagement is less important in the DG as one simply decides how much one would like to give up.

Because OT facilitates positive social behaviors in a variety of mammal species [31], the impact on generosity found in humans is not unexpected. But we were surprised by the 80% increase in generosity OT induced in a setting that precluded face-to-face interactions. For comparison, a related study of interpersonal trust showed only a 17% increase in DM1 monetary transfers to a stranger in the “trust game” for those given 24 IU intranasal OT compared to those given a placebo [37]. OT appeared to have selectively affected the understanding of how another would experience a negative emotion, and seemed to have motivated a desire to reduce DM2s' experienced negativity. This could be called empathy.

Based on a large number of experimental studies, Batson has proposed the empathy-altruism hypothesis in which feeling empathy for another provokes a desire to help him or her [20]. The findings here support and extend this proposal into what might be called the empathy-generosity hypothesis. When induced to take another's perspective in the UG, due to the risk of punishment, transfers are higher than in the DG. We showed that empathy is a likely causal factor because OT infusion produced an increase in generosity.

There are several possible reasons besides empathy that offers in the UG were more generous by those given OT, though these appear unlikely. First, previous studies have shown that those on OT are not cognitively impaired [37], [50]. Indeed, if this were the case, one would expect that the offers in the DG and the punishment threshold in the UG would vary between those on OT and placebo. Second, OT could have increased risk aversion; that is, DM1s on OT may have made larger UG offers to avoid the chance they would be rejected. The lack of a difference in the rejection threshold between the OT and placebo groups indicates this is implausible. Further, a control task involving financial risk-taking in a related experiment showed that risk aversion was unaffected by OT [37]. Lastly, our results are unlikely to be due to the small stakes involved. Increasing the stakes in the UG does not have a substantive effect on DM1 offers or DM2 punishment thresholds [51].

Generosity may be part of the human repertoire to sustain cooperative relationships [19]. Several neural mechanisms likely support generosity. OT can induce dopamine release in ventromedial regions associated with reward [52] reinforcing generosity. A recent fMRI study of donations to charities [28], showed increased activation in the subgenual region of the cingulate cortex (Brodmann area 25) when making a charitable donation compared to receiving a monetary reward. The subgenualis is dense in OT receptors and modulates striatal dopamine release [53]–[54]. OT also down-regulates amygdala activity in humans [55], potentially reducing anxiety associated with relinquishing resources. OT has also been shown to increase the ability to intuit people's intentions from facial expressions [56]. Although choices in the present experiment were made by computer, OT might enhance the recognition of autonomic emotional displays associated with generosity during face-to-face interactions. We did not find evidence that those given OT were more emotionally labile (using the Affective Intensity Measure [57], p = .74) nor had greater attachment anxiety or avoidance of others via the Experiences in Close Relationships-Revised questionnaire [58], (p = .81, p = .75, respectively).

Although we artificially raised OT levels in this study to establish a causal mechanism producing generosity, OT can be enhanced nonpharmacologically in a variety of ways, including touching, safe environments, and receiving a signal of trust from another person [31], [34]. By increasing OT the ability to empathize with others, and the motivation to be generous with them, are enhanced. Indeed, mice that lack OT receptors suffer from social amnesia [59]. This suggests that a variety of factors we encounter in our daily lives may motivate us to be generous—even with strangers.

## Materials and Methods

This experiment was approved by the Institutional Review Boards of UCLA and Claremont Graduate University. All participants gave written informed consent prior to inclusion. There was no deception in any part of this experiment, and we maintained a double-blind design. A total of 68 males participated in the experiment with 34 of them receiving OT and 34 receiving placebo. The mean age was 21.8 (SD 3.8). We recruited only male subjects because of the possibility of an unintended miscarriage in females as well as the varying effects of OT over the menstrual cycle. All subjects were given a screening by a licensed medical doctor (S.A.) for possible contraindications for OT. No adverse events occurred. Exclusion criteria included significant medical or psychiatric illness, medications that interact with OT, and drug or alcohol abuse.

Participants were infused by nasal inhaler with either 40 IU of OT or normal saline of the same amount. Following published pharmacokinetics [60], the OT was allowed to load for 60 minutes prior to the UG and DG choices. After substance administration, participants completed questionnaires by computer to measure demographic, social, and psychological traits. There were no significant differences between the OT and placebo groups in the questionnaire responses.

After the waiting period, participants read self-paced instructions while they sat in partitioned computer stations. The instructions emphasized that the games were one-shot. After the instructions, participants were prompted verbally to ask questions prior to commencement of the games. Choices in the UG and DG were made by computer. Participants were randomly assigned to dyads by proprietary software. All participants were asked to make choices as DM1 and DM2 in the UG, and as DM1 in the DG. Payoffs were determined by randomly assigning participants to be either DM1 or DM2 in the UG, and as DM1s in the DG. After the experiment, participants were paid their earnings in private by a lab administrator.
